# HT-Fed-GAN: Federated Generative Model for Decentralized Tabular Data Synthesis

**DOI:** 10.3390/e25010088

**Published:** 2022-12-31

**Authors:** Shaoming Duan, Chuanyi Liu, Peiyi Han, Xiaopeng Jin, Xinyi Zhang, Tianyu He, Hezhong Pan, Xiayu Xiang

**Affiliations:** 1 School of Computer Science, Harbin Institute of Technology (Shenzhen), Shenzhen 518055, China; 2 Insititute of Data Security, Harbin Institute of Technology (Shenzhen), Shenzhen 518055, China; 3 Peng Cheng Laboratory, Department of New Networks, Shenzhen 518000, China; 4 College of Big Data and Internet, Shenzhen Technology University, Shenzhen 518100, China; 5 School of Computer Science and Technology, The Chinese University of Hong Kong, Shenzhen 518172, China

**Keywords:** privacy-preserving data synthesis, federated learning, federated generative model, decentralized tabular data synthesis, Gaussian mixture model

## Abstract

In this paper, we study the problem of privacy-preserving data synthesis (PPDS) for tabular data in a distributed multi-party environment. In a decentralized setting, for PPDS, federated generative models with differential privacy are used by the existing methods. Unfortunately, the existing models apply only to images or text data and not to tabular data. Unlike images, tabular data usually consist of mixed data types (discrete and continuous attributes) and real-world datasets with highly imbalanced data distributions. Existing methods hardly model such scenarios due to the multimodal distributions in the decentralized continuous columns and highly imbalanced categorical attributes of the clients. To solve these problems, we propose a federated generative model for decentralized tabular data synthesis (HT-Fed-GAN). There are three important parts of HT-Fed-GAN: the federated variational Bayesian Gaussian mixture model (Fed-VB-GMM), which is designed to solve the problem of multimodal distributions; federated conditional one-hot encoding with conditional sampling for global categorical attribute representation and rebalancing; and a privacy consumption-based federated conditional GAN for privacy-preserving decentralized data modeling. The experimental results on five real-world datasets show that HT-Fed-GAN obtains the best trade-off between the data utility and privacy level. For the data utility, the tables generated by HT-Fed-GAN are the most statistically similar to the original tables and the evaluation scores show that HT-Fed-GAN outperforms the state-of-the-art model in terms of machine learning tasks.

## 1. Introduction

With the development of digital technology, tabular data, such as electronic health records (EHR) or financial data, have been increasingly collected and used. The data types are each owned by different parties and horizontally partitioned between multiple parties. Data holders sometimes intend to publish their data for various purposes such as academic research [1], service improvements [2], or public competitions [3]. Consider a scenario of data publishing as follows: There are several hospitals that have some data on different individuals with the same disease. A single hospital has a small amount of data, which it hopes to jointly release to a third party for machine learning tasks such as predicting heart disease. However, considering the privacy and security concerns, the hospital does not want to jeopardize the privacy of the individuals contained in the released data. In the era of big data, similar scenarios have become more and more common. Thus, there is a strong motivation to develop privacy-preserving data-publishing (PPDP) methods for distributed tabular data.

In recent years, privacy-preserving data synthesis (PPDS) [4,5,6,7] as a promising method of solving the problem of PPDP has been well studied. PPDS builds a generative model to synthesize privacy-preserving synthetic data from private data and protects privacy by sharing synthetic data instead of real data.

**Figure 1:**
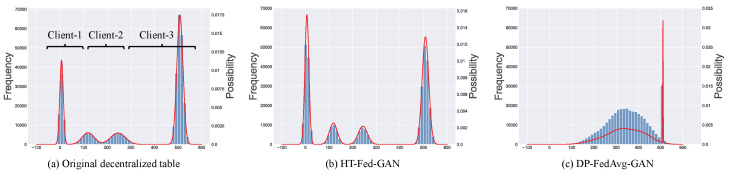
Multimodal distribution in decentralized tables with an example of a count column from the Intrusion dataset [15]. Blue bars indicate the frequency of numerical values and red lines represent the modes in the data. (**a**) Original decentralized table. (**b**) Synthetic table of our method. (**c**) Synthetic table of DP-FedAvg-GAN [8].

Some previous studies [8,9,10,11] have trained a generative model in a decentralized setting on decentralized datasets under the federated learning framework. However, the existing federated generative models are designed for image or text data and not for tabular data. Unlike images, tabular data usually contain a mix of discrete and continuous columns and real-world datasets with highly imbalanced data distributions. Directly applying existing federated generative models to model decentralized tabular data will suffer from two limitations. First, the multimodal distributions in the continuous columns across the clients will lead to mode collapse [12,13] (as shown in Figure 1c), which is called the multiple-mode synthesis problem. In a centralized setting, the state-of-the-art tabular GAN [12] augments the training input using multimodal distributions to eliminate mode collapse. Unfortunately, global multimodal distributions cannot be obtained in federated learning due as there is no global overview of the data statistics. As shown in Figure 1a, there are four modes in the original data distributed among three clients. The state-of-the-art federated image GAN [8] applied to the decentralized tables experiences mode collapse, as shown in Figure 1c. As we can see, the data synthesized by the DP-FedAavg-GAN [8] has only one or two modes and there is a big gap between the synthetic data and the original data in terms of data distribution. Second, the highly imbalanced categorical columns in the decentralized tables further increase the gap between the synthetic data and the real data. On the one hand, the minor categories will be ignored by the model without sufficient training opportunities [12]. On the other hand, the highly imbalanced categories among the clients lead to the weight divergence of a local discriminator, making the discriminator aggregating more difficult [14].

To address these challenges, in this paper, a novel solution for privacy-preserving decentralized tabular data synthesis is proposed called HT-Fed-GAN. In HT-Fed-GAN, a federated variational Bayesian Gaussian mixture model (Fed-VB-GMM) is designed to learn the global multimodal distributions from decentralized continuous columns and then augment the representation according to the learned modes, which can effectively eliminate the mode collapse problem. Then, federated conditional one-hot encoding is used to encode the categorical columns and the conditional sampling method is proposed to rebalance the imbalanced categorical attributes across the clients. Finally, a privacy consumption-based federated conditional GAN is used to model the decentralized tables.

For our experiments, we evaluate HT-Fed-GAN with the state-of-the-art federated generative model on five real-world datasets. The experimental results show that the data generated by HT-Fed-GAN are statically similar to real-world data. For the machine learning tests, our method outperforms the state-of-the-art model. Furthermore, we customize the membership inference attack method presented in [16] to attack HT-Fed-GAN. The experimental results show that HT-Fed-GAN achieves the best trade-off between the privacy level and data utility.

The main contributions of our paper are as follows:

1. We propose HT-Fed-GAN, a novel, practical federated conditional generative approach for synthesizing decentralized tabular data. To the best of our knowledge, this is the first work on privacy-preserving data synthesis on decentralized tabular datasets.
2. We propose the federated variational Bayesian Gaussian mixture model for extracting the multimodal distributions from decentralized tables without disclosing data, which can effectively eliminate mode collapse.
3. We propose the federated conditional one-hot encoding and conditional sampling method for highly imbalanced categorical columns, which preserves the real distributions of the categorical attributes.
4. We conduct experiments on five real-world datasets and the results demonstrate that HT-Fed-GAN offers desirable data with a high privacy level.

The rest of this paper is organized as follows. In Section 2, we discuss the related works. Then, in Section 3, we describe the preliminaries of our HT-Fed-GAN. Then, we introduce the proposed HT-Fed-GAN in Section 4 and evaluate the proposed HT-Fed-GAN in Section 5. Finally, we conclude this paper in Section 6.

## 2. Related Works

Research on privacy-preserving data synthesis has received widespread attention recently. A promising method is using GAN to synthesize fake data similar to real-world data [4,8,17]. For tabular data, medGAN [17] uses a pre-trained auto-encoder to convert discrete values into a continuous representation. The decoder of the auto-encoder translates the continuous output of the generator into the format of the real-world tabular data. TableGAN [4] adds a classifier based on DCGAN to improve the quality of the synthetic table and the privacy penalty in the loss function for data privacy. CTGAN [12] is a state-of-the-art work for synthesizing tabular data, which proposes mode-specific normalization, a conditional generator, and training using sampling strategies to solve the problems of multiple modes in continuous columns and categorical imbalances in discrete columns of tabular data. These studies have been successfully applied to the synthesis of tabular data in centralized settings but failed in decentralized scenarios.

In a decentralized setting, there are no GAN-based methods for tabular data. Federated GANs, such as MD-GAN [18] and FeGAN [19,20], train a federated GAN on the distributed data. However, these methods are vulnerable to privacy attacks [16]. To protect data privacy, DP-FedAvg-GAN [8] and FL-GAN [21] introduce differential privacy [22] into the training process. Unfortunately, these methods cannot be applied to tabular data directly because the problems of multiple modes in continuous columns and categorical imbalances in discrete columns lead to model collapse.

## 3. Preliminaries

In this section, we describe the background of our proposed methods. First, we describe the variational Bayesian Gaussian mixture model (VB-GMM). This algorithm is the basis of the Fed-VB-GMM. Second, we introduce the differential privacy (DP) technique. This technique is the basic technology of the proposed privacy consumption-based federated conditional GAN. Finally, we present the membership inference attack. This attack method is used to verify the privacy level of our HT-Fed-GAN. Table 1 summarizes the frequently used notations in this paper.

### 3.1. Variational Bayesian Gaussian Mixture Model

**Figure 2:**
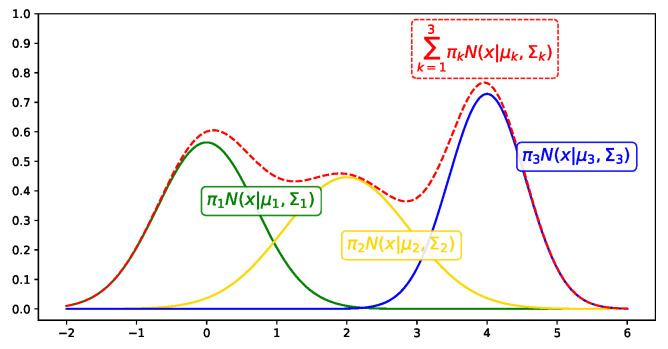
Example of a GMM. The red line represents the GMM and each colored line represents a Gaussian distribution.

The Gaussian mixture model (GMM) is a probabilistic model, as shown in Equation (1).

(1) $$p\left(x|\pi ,\mu ,\Sigma \right)=\sum_{k=1}^{K}\pi_{k}N\left(x|\mu_{k},\Sigma_{k}\right)$$

where $\pi_{k}$ represents the mixture weight of the *k*-th Gaussian distribution and $N(x|\mu_{k},\Sigma_{k})$ represents the *k*-th Gaussian distribution with mean $\mu_{k}$ and variance $\Sigma_{k}$. The GMM is represented as the sum of the number of *K* Gaussian distributions multiplied by the corresponding weight $\pi_{k}$. Figure 2 shows an example of a GMM with three clusters [23]. There are four parameters π,μ,Σ, and *K* to be optimized when the GMM is applied to a dataset.

The variational Bayesian Gaussian mixture model (VB-GMM) [24] is a model that applies the variational Bayesian method to estimate the parameters of the GMM [23]. Three distributions, Dirichlet, Gaussian, and Wishart, are introduced to be the prior distributions for π,μ, and Λ (inverse matrix of Σ), in the VB-GMM algorithm, respectively. These three distributions are shown in Equations (2)–(4).

(2) π∼Dir(α)

(3) $$\mu ∼N(m,{\left(\beta \Lambda \right)}^{-1})$$

(4) W(W,ν)

where α is the parameter of the Dirichlet distribution, which represents the prior distribution of π; *m* and β are the parameters of the Gaussian distribution, which represent the prior distribution of μ; and *W* and ν are the parameters of the Wishart distribution, which represent the prior distribution of Λ. The parameters of the GMM can be calculated using the variational EM algorithm [23,24,25].

### 3.2. Differential Privacy

Differential privacy [22,26] is a mathematical algorithm that indicates the level of privacy protection of individuals in a dataset.

**Definition** **1** (Neighboring Dataset). *Two datasets D and $D^{{}^{\prime }}$ are called neighboring datasets if one can be obtained from the other by either adding or removing one record. This is denoted as*

(5) $$\exists x\in D,\;D\backslash \left\{x\right\}=D^{{}^{\prime }}$$

**Definition** **2** (Differential Privacy [26]). *A randomized algorithm A has (ϵ,δ)-differential privacy if for any output subset S and two neighboring datasets D and $D^{{}^{\prime }}$*

(6) $$P\left(A\left(D\right)\in S\right)\leq e^{\epsilon }\cdot P\left(A\left(D^{{}^{\prime }}\right)\in S\right)+\delta$$

*where A(D) and $A(D^{{}^{\prime }})$ are the outputs of the algorithm for the neighboring datasets D and $D^{{}^{\prime }}$, respectively, and P is the randomness of the noise in the algorithm.*

In practice, we employ a randomized mechanism *A* that ensures DP for any deterministic vector-valued computation function *h*: $D\to R^{n}$. More specifically, mechanism *A* covers the sensitivity of *h* by injecting noise into the output of *h*. The sensitivity measures the maximum degree of variation in any pair of inputs *d* and $d^{{}^{\prime }}$.

**Definition** **3** (Sensitivity). *The sensitivity of a function h for any pair of inputs $d,d^{{}^{\prime }}\in J$ is*

(7) $${△}_{h}=\underset{d,d^{{}^{\prime }}}{max}{\left\|h\left(d\right)-h\left(d^{{}^{\prime }}\right)\right\|}_{2}$$

*where ${\left\|.\right\|}_{2}$ represents the $l_{2}$-norm. Based on the sensitivity of h, we can design the degree of noise to achieve local differential privacy.*

The post-processing of differential privacy allows us to impose differential privacy on the discriminator and the generator will have the same level of privacy as the discriminator. The proof of the post-processing can be found in [26].

**Theorem** **1** (Post-processing). *Let A:D→S be an (ϵ,δ)-differential privacy algorithm and an arbitrary randomized mapping $f:S\to S^{{}^{\prime }}$, then, $f\circ A:D\to S^{{}^{\prime }}$ has (ϵ,δ)-differential privacy.*

### 3.3. Membership Inference Attack

A membership inference attack [27] is a new attack method against machine learning models, which mainly infers the target samples in the training datasets according to the output of a target machine learning algorithm. It is assumed that an attacker has black-box access to the target model and knows the details of the target model. However, in PPDP, the attacker can only obtain the shared data and knows nothing about the target model. Therefore, we adopt the membership inference attack method presented in [16] to attack our HT-Fed-GAN in this paper. The attacker uses the fake data generated by the target generation model to train a GAN model. The discriminator of the trained GAN is used as an attack model to infer whether the data held by the attacker is the training sample of the target model.

## 4. HT-Fed-GAN

**Figure 3:**
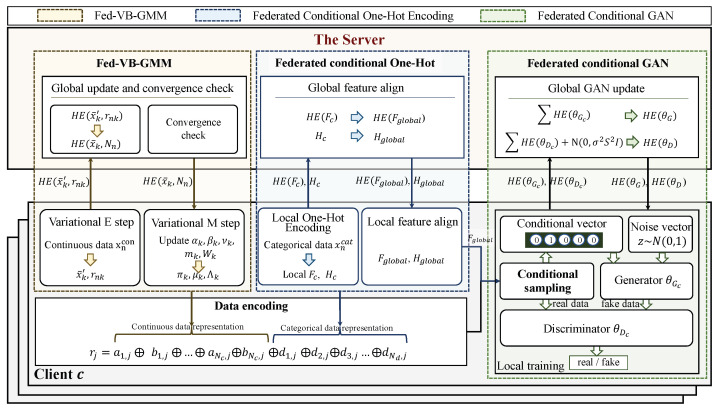
The framework of HT-Fed-GAN. The pipeline of HT-Fed-GAN consists of two steps: data encoding and federated GAN training. In the data encoding process, each sample in the decentralized tables is encoded as $r_{j}$ by the proposed Fed-VB-GMM and the federated conditional one-hot encoding method. Then, in the federated GAN training process, the conditional sampling method and a privacy consumption-based federated conditional GAN training algorithm are designed to model the decentralized tabular data.

As shown in Figure 3, the pipeline of the HT-Fed-GAN mainly consists of two steps: data encoding and federated GAN training.

1. *Data Encoding.* To eliminate the multiple-mode synthesis problem, the federated variational Bayesian Gaussian mixture model (Fed-VB-GMM) (Algorithm 1) is designed to learn the multimodal distributions in continuous columns from each client. Then, the continuous columns are encoded to become input representations using Equation (24), which uses the extracted multimodal distributions as prior knowledge. In addition, a federated conditional one-hot encoding method is proposed to encode the discrete columns using Equation (25). Finally, each sample in the decentralized tables is represented as the representation shown in Equation (26).
2. *Federated GAN Training*. To alleviate the problem of categorical imbalances across the clients, a conditional sampling method is proposed to rebalance the categorical distributions during federated training. In addition, to prevent the privacy leakage caused by the federated conditional GAN, a privacy consumption-based federated conditional GAN training algorithm (Algorithm 2) is presented to flexibly control the privacy level of GAN.

### 4.1. Federated Variational Bayesian Gaussian Mixture Model

In a centralized setting, the state-of-the-art model [12] performs the VB-GMM [24] to learn a GMM in a continuous column and then perform a mode-specific normalization in this column, which can effectively eliminate mode collapse. However, there is no global overview of data statistics in federated learning and the multimodal distributions cannot be learned further.

Therefore, we propose Fed-VB-GMM to extract multimodal distributions in continuous columns from clients. As shown in Algorithm 1, we initialize all parameters randomly on the server and then send them to each client (Step 1 of Algorithm 1). Fed-VB-GMM consists of two steps: the variational E step and the variational M step. The purpose of the variational E step is to estimate the responsibilities $r_{nk}$, which indicates the probability that data belong to the *k*-th mode. First, we estimate the mixture weight π and precision Λ, as shown in Equations (8) and (9). ψ(.) in Equation (8) represents the digamma function [28]. Then, we calculate the responsibilities $r_{nk}$ (Equation (10)) and the mean of the *k*-th mode, which is marked as ${\bar{x}}_{k}^{{}^{\prime }}$ (Equation (11)), locally because they need private data. Finally, each client encrypts $r_{nk}$ and ${\bar{x}}_{k}^{{}^{\prime }}$ using homomorphic encryption [29] and then sends them to the server.
**Algorithm 1:** Fed-VB-GMM Algorithm**Input**: DataSet $X=\{x_{1},...,x_{n}\}$**Output**: α,m,β,Λ,W,ν**Step 1 (Initialization): Server**Initialize α,m,β,Λ,W,ν,K randomlySend α,m,β,Λ,W,ν,K to each client**Step 2 (Variational E step): Client**Get α,m,β,Λ,W,ν,K from serverEstimate π,Λ
(8)$$ln\tilde{\pi }=\psi \left(\alpha_{k}\right)-\psi \left(\sum_{i=1}^{K}\left(\alpha_{i}\right)\right)$$
(9)$$ln\tilde{\Lambda }=\sum_{d=1}^{D}\psi \left(\frac{\nu_{k}+1+d}{2}\right)+Dln2+ln\left|W_{k}\right|$$Estimate $r_{nk}$ by π and Λ
(10)$$\begin{array}{cc} r_{nk}= & \pi_{k}{\left|\Lambda_{k}\right|}^{1/2}\times \\ \; & exp\{-\frac{D}{2\beta_{k}}-\frac{\nu_{k}}{2}{\left(x_{n}-m_{k}\right)}^{T}W_{k}\left(x_{n}-m_{k}\right)\} \end{array}$$Estimate local ${\bar{x}}_{k}^{{}^{\prime }}$
(11)$${\bar{x}}_{k}^{{}^{\prime }}=\sum_{n=1}^{N_{client}}r_{nk}x_{n}$$Encrypt $r_{nk}$ and ${\bar{x}}_{k}^{{}^{\prime }}$ using homomorphic encryption and send to server**Step 3 (Variational M step): Server**Get $r_{nk}$ and ${\bar{x}}_{k}^{{}^{\prime }}$ from each clientCompute $N_{k}$ by $r_{nk}$ from each client c ∈C
(12)$$N_{k}=\sum_{c\in C}^{C}r_{nk}$$Update ${\bar{x}}_{k}$ by ${\bar{x}}_{k}^{{}^{\prime }}$ from each client c ∈C
(13)$${\bar{x}}_{k}=\frac{1}{N_{k}}\sum_{c\in C}^{C}{\bar{x}}_{k}^{{}^{\prime }}$$Update $\alpha_{k},\beta_{k},\nu_{k}$, $m_{k}$
(14)$$\alpha_{k}=\alpha_{0}+N_{k}$$
(15)$$\beta_{k}=\beta_{0}+N_{k}$$
(16)$$\nu_{k}=\nu_{0}+N_{k}$$
(17)$$m_{k}=\frac{1}{\beta_{k}}\left(\beta_{0}m_{0}+N_{k}{\bar{x}}_{k}\right)$$Send ${\bar{x}}_{k},N_{k},\alpha_{k},\beta_{k},\nu_{k}$ to each client**Step 4 (Variational M step): Client**Each client decrypts and updates ${\bar{x}}_{k},N_{k},\alpha_{k},\beta_{k},\nu_{k}$Compute $S_{k}^{{}^{\prime }},L^{{}^{\prime }},W_{k}^{{}^{\prime }}$ in the client
(18)$$S_{k}^{{}^{\prime }}=\sum_{n=1}^{N_{c}}r_{nk}\left(x_{n}-{\bar{x}}_{k}\right){\left(x_{n}-{\bar{x}}_{k}\right)}^{T}$$
(19)$$\begin{array}{cc} L^{{}^{\prime }}= & -\sum_{n=1}^{N_{c}}\sum_{k=1}^{K}\left(e^{r_{nk}}\times r_{nk}\right)\\ \; & -\sum_{k=1}^{K}\left(\frac{\nu_{k}Dln2}{2}-\sum_{k=1}^{K}ln\Gamma \left(\nu_{k}\right)\right)\\ \; & -(ln\Gamma \left(\sum_{k=1}^{K}\alpha_{k}\right)-\sum_{k=1}^{K}ln\Gamma \left(\alpha_{k}\right))\\ \; & -\frac{D\sum_{k=1}^{K}ln\beta_{k}}{2} \end{array}$$
(20)$$W_{k}^{-1^{\prime }}=W_{0}^{-1}+\frac{\beta_{0}N_{k}}{\beta_{0}+N_{k}}\left({\bar{x}}_{k}-m_{0}\right){\left({\bar{x}}_{k}-m_{0}\right)}^{T}$$Encrypt $S_{k}^{{}^{\prime }},L^{{}^{\prime }},W_{k}^{{}^{\prime }}$ and send to server**Step 5 (Convergence Check): Server**Get $S_{k}^{{}^{\prime }},L^{{}^{\prime }},W_{k}^{{}^{\prime }}$ from each clientUpdate $S_{k}$ by $S_{k}^{{}^{\prime }}$ from each client c ∈C
(21)$$S_{k}=\frac{1}{N_{k}}\sum_{c\in C}^{C}S_{k}^{{}^{\prime }}$$Update $W_{k}$ by $W_{k}^{{}^{\prime }}$ and $S_{k}$
(22)$$W_{k}^{-1}=W_{k}^{{}^{\prime }}+N_{k}S_{k}$$Compute lower bound *L* by $L^{{}^{\prime }}$ and $W_{k}$
(23)$$L=L^{{}^{\prime }}-\sum_{k=1}^{K}(\nu_{k}|W_{k}\left|\right)$$Compare the lower bound *L* in this iteration with the last $L_{last}$. If the difference between them is less than a predefined value ϵ, the process ends. Otherwise, return to Step 2.

In the variational M step, we compute some parameters according to the updated parameters in the variational E step. We update $N_{k}$, ${\bar{x}}_{k}$, $\alpha_{k},\beta_{k},\nu_{k}$, and $m_{k}$, as shown in Equations (12)–(17), where $N_{k}$ and ${\bar{x}}_{k}$ represent the global responsibility and the mean of the *k*-th mode, respectively. We found that the covariance matrix of the *k*-th mode $S_{k}$ requires private data and $W_{k}$ and *L* are hardly computed under homomorphic encryption, where *L* represents the lower bound of the Gaussian mixture model (GMM). To update these three parameters, we compute the complex part on the client side (Equations (18)–(20) and then merge them on the server (Equations (21)–(23)). Γ(.) in Equation (19) represents the gamma function [30]. We compare the lower bound of this iteration with that of the previous round; if the difference between them is less than a predefined value ϵ, the process ends. Otherwise, return to Step 2.
**Algorithm 2:** Training Algorithm of HT-Fed-GAN
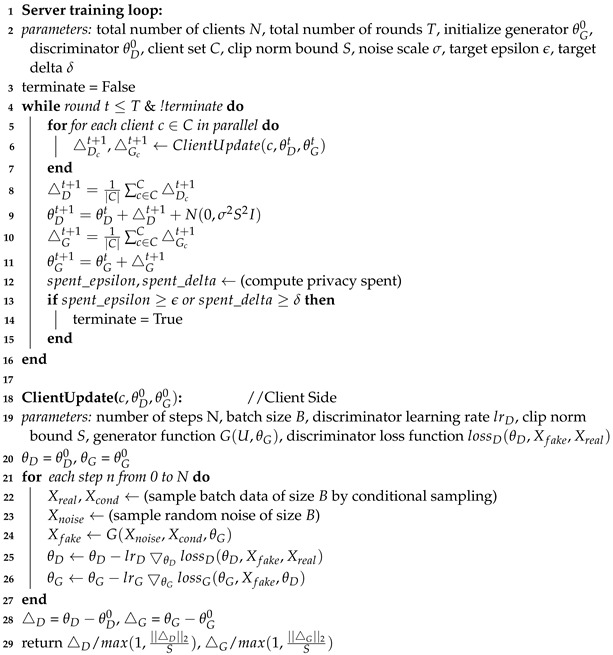


    When the algorithm converges, we use the extracted global Gaussian mixture model to encode the continuous columns as follows:

(24) $$r_{j}^{con}=a_{1,j}⊕b_{1,j}⊕\ldots ⊕a_{N_{c},j}⊕b_{N_{c},j}$$

where $a_{i,j}=\frac{c_{i,j}-\eta_{k}}{4\phi_{k}}$, $c_{i,j}$ is the continuous data; $\eta_{k}$ and $\phi_{k}$ are the mean and standard deviation of the *k*-th Gaussian mode, respectively; $b_{i,j}$ represents the one-hot representation of Gaussian mode, which $c_{i,j}$ follows; $N_{c}$ is the number of continuous columns; and ⊕ refers to the concatenate operation.

### 4.2. Federated Conditional One-Hot Encoding and Conditional Sampling

*Federated conditional one-hot encoding.* In general, discrete columns are commonly normalized using the one-hot encoding method. In federated learning, when each client does not expose its data to others, one-hot encoding can only be performed locally. However, since different clients collect data independently, some categories in one client may not be present in other clients. As shown in Algorithm 3, after local one-hot encoding, the local feature header $H_{c}$ and its frequency $F_{c}$ are collected and sent to the server. The server then aligns the features and sends the intersection of the feature header $H_{global}$ back to each client. Meanwhile, the global frequency of each feature $F_{global}$ is computed and sent back to each client. Upon receiving the aligned features, each client creates additional features if necessary and uses zeros to represent the non-presence of the related features. After federated one-hot encoding, the representation of the categorical columns is

(25) $$r_{j}^{cat}=d_{1,j}⊕d_{2,j}⊕\ldots ⊕d_{N_{d},j}$$

where $d_{i,j}$ is a one-hot representation of a categorical value and $N_{d}$ is the number of categorical columns. ⊕ refers to the concatenate operation. The final encoding of the *j*-th record in the tabular data with $N_{c}$ continuous columns and $N_{d}$ categorical columns is

(26) $$r_{j}^{con}⊕r_{j}^{cat}$$

*Conditional sampling*. In the training phase, we propose conditional sampling to rebalance the decentralized categorical attributes. First, we randomly select a categorical column *m* with equal probability. Then, we calculate the probability distribution of each feature *f* in the selected column *m* according to its global frequency $F_{global}\left[m\right]\left[f\right]$. Finally, we sample a feature based on the logarithm of its probability. Compared to random sampling, conditional sampling enables the conditional generator to learn the true global distribution. To represent the above condition for the conditional generator and preserve it in the output, we use a conditional vector [12] to encode the selected category during conditional sampling. Each conditional vector represents a single category.    
**Algorithm 3:** Federated feature-aligning algorithm
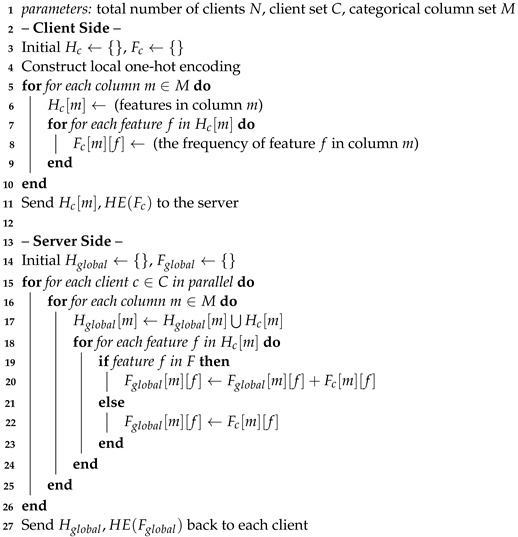


### 4.3. Privacy Consumption-Based Federated Conditional GAN

In HT-Fed-GAN, we customize a federated conditional GAN to model the decentralized tabular data. Existing architectures of decentralized GANs can be classified into two categories: (1) multi-discriminator architectures [8,9], and (2) federated GAN architectures. In the multi-discriminator architecture, multiple discriminators are distributed across the clients and a single generator is trained on the server following the aggregated discriminator. In contrast to the multi-discriminator architecture, both the discriminator and generator are trained locally and aggregated on the server in the federated GAN architecture. The federated GAN architecture has multiple benefits, ranging from better communication overhead, scalability, and training stability compared to the multi-discriminator architecture. Therefore, as shown in Algorithm 2, we adopt the federated GAN architecture as the base architecture of our HT-Fed-GAN.

To prevent the discriminator from memorizing private data during training, we apply user-level differential privacy [26] during the aggregation of the discriminator. Unlike the existing methods [8,9] that use fixed-size federated rounds, where it is difficult to control the privacy level of the generative model, our HT-Fed-GAN introduces privacy consumption as a condition to control the privacy level. We calculate the privacy cost of each federated training round and stop the learning when it meets the pre-defined privacy consumption.

Since tabular data do not have a local structure, we use the fully-connected network in both the generator and discriminator. In the discriminator, the input to the first layer is a vector with a length of 256 that represents one real or fake record. The input of the generator is a latent vector with a length of 128 that is randomly sampled from the unit space. Multiple hidden layers convert the input to a vector with a length of 256, which represents a synthetic record. The dimensions of the input and output of our model are configurable and able to learn from enough attributes. The architecture of the GAN model follows the architecture of CTGAN [12]. We train the model using the objective of WGAN [31] with a gradient penalty.

### 4.4. Privacy Analysis

In this paper, we follow the typical assumption of federated learning, that is, the clients and the server are honest but curious. As shown in Figure 3, the overall pipeline of our HT-Fed-GAN mainly includes two steps: data encoding and federated GAN training. First, during the data encoding process, the Fed-VB-GMM is designed to compute the multimodal distributions of the continuous columns from each client, as shown in Algorithm 1. In addition, the federated conditional one-hot encoding algorithm is proposed to compute the global frequency of the discrete columns from each client, as shown in Algorithm 3. Therefore, the main risk of privacy leakage in the data encoding process is that the statistical information of the clients is leaked during the global distribution calculation. To protect the local private information from being disclosed, the homomorphic encryption [29] technique is used to compute the global statistics under the coordination of the server. Since there are only addition operations on the server, as shown in Algorithm 1 and Algorithm 3, the additively homomorphic encryption scheme in [29] is applied to the HT-Fed-GAN. In this scheme, the participants jointly set up the publish key pk and the secret key sk. The secret key sk is held by each client and kept secret from the server. Each client establishes a different TLS/SSL secure channel to communicate with the server to ensure the integrity of the homomorphic ciphertext.

Second, during the federated GAN training process, HT-Fed-GAN strictly follows the standard federated learning training protocol [11]. Compared with other deep learning models, GAN tends to memorize local training data, which may lead to private information leakage during data synthesis. Thus, we propose a privacy consumption-based federated training method to build a privacy-preserving GAN model. As shown in Algorithm 2, Gaussian noise is injected into the parameters of the discriminator from each client to achieve user-level differential privacy before model aggregation. Following [26], we set the standard deviation of Gaussian noise as $\sigma =\frac{{▵}_{\Sigma }\sqrt{2ln(1.25/\delta )}}{\epsilon }$ to satisfy (ϵ,δ)-differential privacy, where ${▵}_{\Sigma }$ represents the $l_{2}$ sensitivity of the target parameters and ϵ and δ are the privacy budget of the differential privacy. Since the generator’s loss is a function of the discriminator, according to the post-processing property of differential privacy, the generator has the same privacy level as the discriminator. Meanwhile, to control the privacy level of the federated GAN flexibly, the privacy consumption is calculated after each round of federated training and the federated training is stopped until the privacy consumption reaches the privacy budget.

Overall, there is no information leakage during the data encoding process and federated GAN training process in HT-Fed-GAN. Note that the research on the vulnerabilities of federated learning is a hot topic including gradient leakage [32], compromised clients/servers [33], adversarial attacks [34], statistical heterogeneity [35], etc., but these are beyond the scope of this paper.

### 4.5. Membership Inference Attack for HT-Fed-GAN

**Figure 4:**
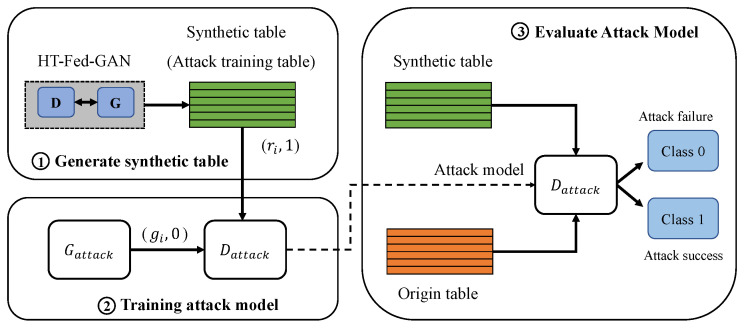
The overall procedure of the customized membership attack method.

To attack the proposed HT-Fed-GAN, we customize the membership inference attack method presented in [16]. In a scenario of releasing data to the public, we attack the entire HT-Fed-GAN model rather than the discriminator [4] because there is no knowledge other than the synthetic data. As shown in Figure 4, the overall procedure of the customized membership inference attack is as follows:

1. Let $M_{target}$ be a target HT-Fed-GAN that an attacker wants to attack. Note that access to the target model $M_{target}$ is not allowed because it is not shared with the public after being trained.
2. Obtain the released synthetic table generated by $M_{target}$, which is the positive training sample for the attack model, as shown in Figure 4. Each record of the synthetic table is denoted as $\left(r_{i},1\right)$, where 1 represents this record in the training table of the target model.
3. We choose ctgan [12] as the attack model and mark it as $M_{attack}$. $G_{attack}$ and $D_{attack}$ represent the generator and discriminator of the attack model, respectively. The discriminator of $M_{attack}$ is used to determine whether the record is the training sample of the target model. The synthetic table generated by $G_{attack}$ is marked as 0, indicating that the data are not a training sample of the target model.
4. Train the attack model using the released synthetic table.
5. Construct the test dataset to evaluate the performance of the attack model. The ratio of real to fake records in the test dataset is 1:1.

## 5. Experiments

In this section, we describe the experimental environments, datasets, and results. We evaluate HT-Fed-GAN on five commonly used machine learning datasets from five domains: personal information, network intrusion, credit cards, forest cover types, and clinical.

### 5.1. Experiment Setup

#### 5.1.1. Environment Setup

We set up our experiment in a distributed computing network equipped with GPUs. There are four nodes in total, one representing the server, and the other three representing clients (Client 1–Client 3) from different organizations in the real world. Each node was configured with an NVIDIA Tesla P100 GPU card. Our code implementation was based on PyTorch [36] 1.4.0, PySyft 0.2.5, Python 3.7.6. For simplicity, we assumed that all clients participated in learning.

#### 5.1.2. Dataset

We used five datasets, as shown in Table 2. Each dataset contained continuous and discrete columns that can be used for machine learning tasks, as follows:

- The Adult [1] dataset comes from the 1994 Census database and contains a lot of personal information (such as income, work hours per week, education, and so on). The income attribute represents the salary for each individual, which is greater than 50 K or less than 50 K. Thus, we performed a binary classification test using this attribute. For the regression tasks, we chose the hours_per_week attribute as the target label, which represents the number of work hours per week.
- The Intrusion [15] dataset comes from a network intrusion detection competition and consists of a wide variety of intrusions simulated in a military network environment. The label attribute represents the type of intrusion, which contains seven classes. Thus, we performed multi-class classification tests using the label attribute. For the regression tasks, we used the count attribute.
- The Credit [37] dataset comes from a Kaggle competition named Credit Fraud Detection and contains transactions made using credit cards in September 2013 by European cardholders. The label attribute indicates whether the record is fraudulent. The amount attribute has the information on the transaction amount. Thus, we performed binary classification and regression tests using the two attributes, respectively.
- The Cover-Type [38] dataset is derived from the US Geological Survey (USGS) and US Forest Service (USFS). The label attribute represents the forest cover type and contains seven classes. The elevation attribute is the elevation of the forest. Thus, we performed multi-class classification and regression tests using this dataset.
- The Health [39] dataset is about cardiovascular diseases (CVDs),and contains 12 features used to predict mortality due to heart failure. The DEATH_EVENT attribute represents whether the patient died during the follow-up period. Thus, we performed a binary classification test using this attribute and used the age attribute for the regression tests.

Table 2 shows the statistical information of the experimental dataset including (from left to right) the dataset, number of records, number of attributes, classification type, attribute for the classification tests, and attribute for the regression tests. We split the original dataset into two parts: a training set (70%) and test set (30%). To construct a biased training subset, we split the training set into three parts as the client dataset based on the split attribute, as shown in Table 2.

#### 5.1.3. Evaluation Method

We evaluated the data synthesis method by comparing the privacy and utility of the synthesized data. The evaluation metrics are detailed below.

- The data-utility-related evaluation metrics are as follows:
  – **Cumulative distributions:** We compared the cumulative distributions for the same attribute between the original data and synthesized data [4]. This mainly compared the statistical similarity between the original data and the synthesized data.
  – **Machine Learning Score:** SDGym [12] is a framework to benchmark the performance of synthetic data generators. We used SDGym to train the machine learning models on the synthetic data and test the trained model on the original dataset. In the training process, the machine learning models and their parameters were fixed in each dataset. We evaluated the performance of the classification tasks using the F1 score (binary classification tasks) or the macro- and micro-F1 scores (multi-class classification tasks) and the regression tasks using the mean absolute error (MAE). For each dataset, we performed four classifiers or regressors to evaluate the performance of the machine learning task.
- The privacy-related evaluation metrics are as follows:
  – **Membership Inference Attack:** We customized the black-box membership inference attack presented in [16] to evaluate the privacy of our HT-Fed-GAN without any auxiliary information other than the synthetic data. The detailed procedure is described in Section 4.5.

#### 5.1.4. Baseline Model

To the best of our knowledge, there is no federated generative model for decentralized tabular data synthesis. Thus, we used DP-FedAvg-GAN [8] as the baseline model, which is the state-of-the-art work for synthesizing decentralized images. To generate the tabular data, we customized DP-FedAvg-GAN to model the structured data.

#### 5.1.5. Parameter Setup

There are two parameters ϵ and δ to control the level of privacy in HT-Fed-GAN. Following [4], there are two types of privacy levels in HT-Fed-GAN: the low-privacy setting and high-privacy setting. The low-privacy setting indicates that differential privacy was not applied to the federated GAN training process, whereas the high-privacy setting denotes that differential privacy was used in the federated GAN training process. In this paper, in the high-privacy setting, we set the privacy budget as ϵ = 0.5 and δ = 9.8. The values of the privacy budgets in DP-FedAvg-GAN [8] were the same as those used in HT-Fed-GAN.

### 5.2. Cumulative Distributions

**Figure 5:**
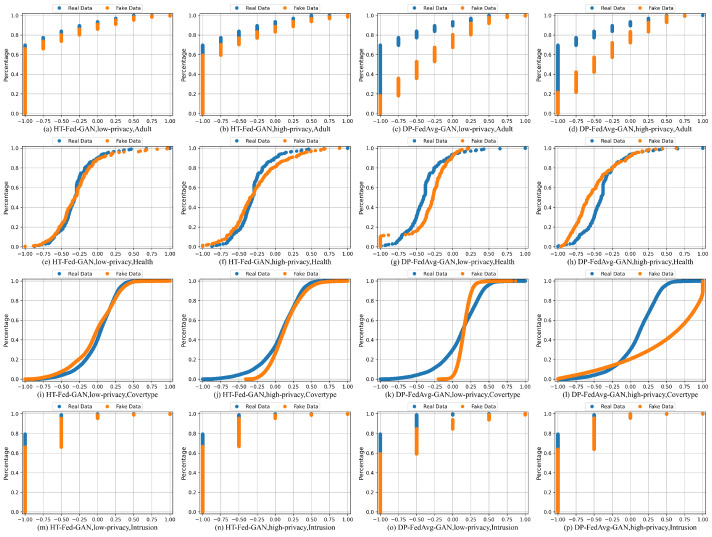
Cumulative distributions. Cumulative distributions of attributes (workclass, platelets, elevation, and label for each dataset, respectively) by HT-Fed-GAN with low-privacy setting, high-privacy setting, and DP-FedAvg-GAN. Blue lines represent the original attributes and orange lines represent the synthetic data. The low-privacy setting indicates that differential privacy was not used in HT-Fed-GAN, whereas the high-privacy setting denotes that differential privacy was used in HT-Fed-GAN and the privacy budgets were ϵ = 0.5 and δ = 9.8.

Figure 5 presents the cumulative distributions of several attributes from the four datasets: Adult (work class), Health (platelets), Cover-Type (elevation), and Intrusion (label). For the Credit dataset, no matter how hard we tried, DP-FedAvg-GAN experienced mode collapse. Therefore, we did not show the statistical comparison of the Credit dataset. In Figure 5, the orange lines represent the synthetic data and the blue lines are the real values in the original data.

Figure 5a–d,m–p are the cumulative distributions of the categorical columns. For the categorical columns, the cumulative distributions of HT-Fed-GAN were mostly close to the original tables, which means that our HT-Fed-GAN can capture the real distributions of the highly imbalanced categorical attributes among the clients. Figure 5e–l show the cumulative distributions of the continuous columns. HT-Fed-GAN shows a more realistic statistical distribution than DP-FedAvg-GAN, which demonstrates that HT-Fed-GAN is superior to DP-FedAvg-GAN in terms of synthesizing the multimodal distributions in decentralized continuous columns.

To summarize, HT-Fed-GAN with the low-privacy setting showed the best synthesis performance. In almost all the datasets, the synthetic data of HT-Fed-GAN were statistically similar to the real data. DP-FedAvg-GAN showed the worst performance because it was not designed for decentralized tabular data.

### 5.3. Machine Learning Scores

In this section, we use several classification and regression algorithms to evaluate the quality of the synthetic data.

#### 5.3.1. Binary Classification

**Figure 6:**
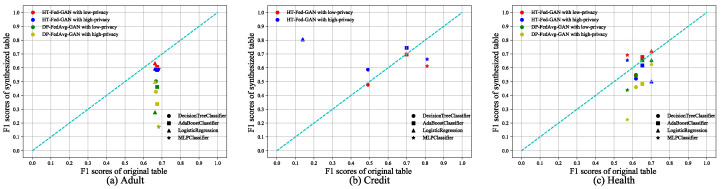
Binary classification tests. The binary classification scores of HT-Fed-GAN and DP-FedAvg-GAN. The x-axis of each figure is the F1 score of the classifier trained using the original table. The y-axis is the F1 score of the classifier trained using the synthesized table. The low-privacy setting indicates that differential privacy was not used in HT-Fed-GAN, whereas the high-privacy setting denotes that differential privacy was used in HT-Fed-GAN and the privacy budgets were ϵ = 0.5 and δ = 9.8.

We compared HT-Fed-GAN and DP-FedAvg-GAN on the Adult, Credit, and Health datasets, respectively. We used the AdaBoost, decision tree, logistic regression, and multi-layer perceptron classifiers and their recommended parameter settings from the scikit-learn web pages to test the binary classification tasks. The experimental results are shown in Figure 6, where the x-axis represents the F1 score of the classifier trained using the original tables and the y-axis is the F1 score of the classifier trained using the synthetic tables. The diagonal line indicates that the performance of the classifiers trained on the synthetic data is the same as that on the real-world data. Table 3 shows the average F1 scores of the classifiers trained on each synthetic table and the original table.

From Figure 6 and Table 3, we can see that HT-Fed-GAN outperformed DP-FedAvg-GAN in terms of the binary classification tests. Figure 6a shows the performance on the Adult dataset and the average F1 score of HT-Fed-GAN exceeds that of DP-FedAvg-GAN by 25% with the low-privacy setting and 23% with the high-privacy setting. Figure 6b shows the results on the Credit dataset, where DP-FedAvg-GAN experienced mode collapse no matter how hard we tried. Because on the Credit experiment the data of each client were non-IID, as shown in Table 2, this resulted in mode collapse during the training process of DP-FedAvg-GAN. Surprisingly, there were some classifiers trained on the data generated by HT-Fed-GAN that performed better than those trained on the original data, as shown in Figure 6b,c. This means that our HT-Fed-GAN not only maintained a similar distribution to the real data but also extracted some prominent features that were helpful for machine learning tasks.

#### 5.3.2. Multi-Classification

**Figure 7:**
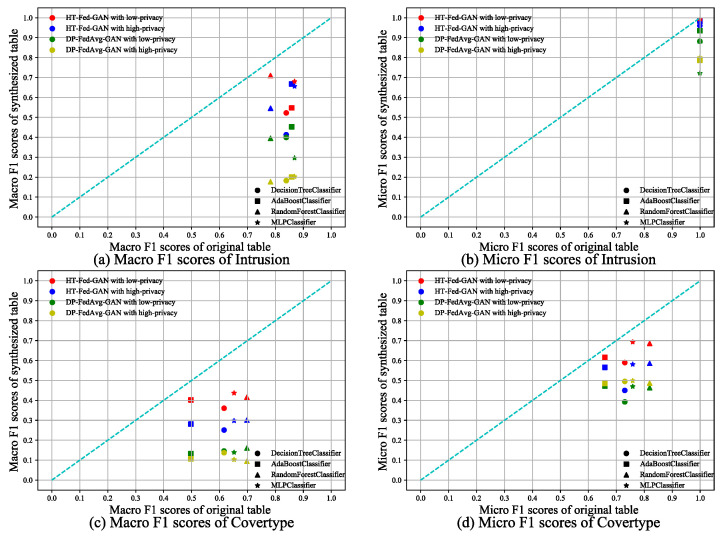
Multi-classification tests. The multi-classification scores of HT-Fed-GAN and DP-FedAvg-GAN. The x-axis of each figure is the macro/micro-F1 scores of the classifier trained with the original table. The y-axis is the macro/micro-F1 scores of the classifier trained with the synthesized table. The low-privacy setting indicates that differential privacy was not used in HT-Fed-GAN, whereas the high-privacy setting denotes that differential privacy was used in HT-Fed-GAN and the privacy budgets were ϵ = 0.5 and δ = 9.8.

We performed multi-class classification tests on the Intrusion and Cover-Type datasets. Figure 7 shows the experimental results of HT-Fed-GAN compared to DP-FedAvg-GAN. We followed the same plotting method as that shown in Figure 6, which shows the results. Table 4 shows the average macro/micro-F1 scores of the multi-class classification tests.

As shown in Figure 6 and Table 4, in almost all cases, HT-Fed-GAN with the low-privacy setting achieved the best performance. In the two datasets, The average macro-F1 scores of HT-Fed-GAN exceeded those of DP-FedAvg-GAN by 23–28% under the low-privacy setting. This shows that our method outperformed DP-FedAvg-GAN in terms of categorical attribute modeling.

#### 5.3.3. Regression

**Figure 8:**
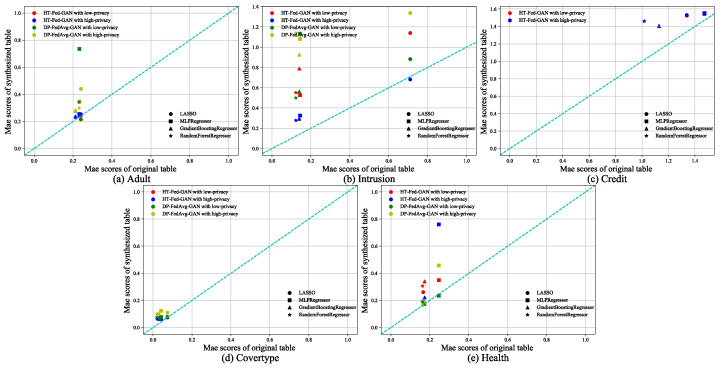
Regression tests. Regression scores of HT-Fed-GAN and DP-FedAvg-GAN. The x-axis of each figure is the MAE score of the regression algorithm trained using the original table. The y-axis is the MAE score of the regression algorithm trained using the synthesized table. The low-privacy setting indicates that differential privacy was not used in HT-Fed-GAN, whereas the high-privacy setting denotes that differential privacy was used in HT-Fed-GAN and the privacy budgets were ϵ = 0.5 and δ = 9.8.

The regression tests followed the same training methods as the classification tests. We used the following four regression algorithms for each dataset: Lasso regression, multi-layer perceptron regression, gradient-boosting regression, and random forest regression. Recall that we used the MAE as the base metric to evaluate the regression models. Figure 8 shows the experimental results of the regression tests. We followed the same plotting method as that used in the classification tests. The diagonal line indicates that the performance of the regression algorithm trained on the synthetic data was equal to that of the real-world data. Table 5 shows the average MAE of the regression tests.

In Figure 8a–d, it can be seen that HT-Fed-GAN outperformed DP-FedAvg-GAN in terms of the MAE score. However, in Figure 8e, it can be seen that HT-Fed-GAN was inferior to DP-FedAvg-GAN both in the low-privacy setting and high-privacy setting. This shows that multimodal distribution augmentation was not dominant in the small datasets. Moreover, Table 5 shows that HT-Fed-GAN with the low-privacy setting had better performance than HT-Fed-GAN with the high-privacy setting, except for the Intrusion dataset.

### 5.4. Results for Privacy

We evaluated our HT-Fed-GAN and the baseline model for privacy using the membership inference attack. For each dataset, we trained four attack models following the procedure described in [16]. Each testing dataset comprised 50% original data labeled as 1 and the rest were synthetic data labeled as 0. We used the F1 score as the metric to evaluate the performance of the attack. If the F1 score was lower than 0.5, it meant that the attack was successful.

Table 6 shows the results of the membership inference attack. HT-Fed-GAN with the high-privacy setting achieved the best performance in defending membership attacks. As the level of privacy increased, the attack performance decreased. We found that the privacy of DP-FedAvg-GAN was similar to that of HT-Fed-GAN in the low-privacy configuration. However, the quality of the synthesized data was far lower than that of HT-Fed-GAN, as described in Section 5.2 and Section 5.3. Therefore, our method showed the best trade-off between the privacy level and utility.

### 5.5. Multimodal Distribution Study

To evaluate the effectiveness of the Fed-VB-GMM in multimodal distribution extractions, we compared the average log-likelihood of the Fed-VB-GMM and VB-GMM on the Adult dataset (age attribute). The average log-likelihood of the data was compared by varying the number of clusters. The average log-likelihood is the degree of likelihood for the probability distribution of the data and is represented by Equation (27).

(27) $$ALL=\frac{-\sum_{i=1}^{N}log\left\{\sum_{k=1}^{K}\pi_{k}N\left(x|\mu_{k},\Sigma_{k}\right)\right\}}{N}$$

**Figure 9:**
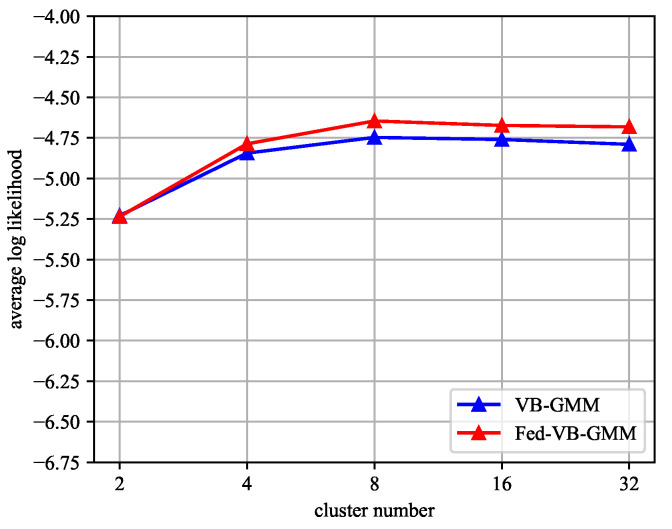
Average log-likelihood of age attribute in the Adult dataset depending on the number of clusters. The blue line represents the VB-GMM and the red line represents the Fed-VB-GMM.

Figure 9 shows the evaluation results. The number of clusters varied from 2 to 32. The blue line represents the VB-GMM and the red line represents the Fed-VB-GMM. Figure 9 shows that the performance of the Fed-VB-GMM was similar to that of the VB-GMM. Of course, there were still some minor differences when the number of clusters increased. These differences were because the homomorphic encryption led to a precision loss during the float-point number computation.

### 5.6. Synthesis Example

Table 7 shows the fake data generated by HT-Fed-GAN with the low-privacy setting on the Adult dataset. Some records of three clients from the original dataset are shown in Table 8 after choosing a subset of columns for space consideration. In Table 8, rows 1–2 are from Client 1, rows 3–4 from Client 2, and the last two rows are from Client 3. As shown, there is no one-to-one relationship between Table 8 and Table 7, and the real records have very different values from the synthesized records. It is almost impossible to re-identify the original information from the synthesized data.

## 6. Conclusions

In this paper, we studied the problem where existing solutions suffer from mode collapse and private leakage from membership inference attacks on privacy-preserving data synthesis (PPDS) for tabular data in a distributed multi-party environment. We propose HT-Fed-GAN, a novel federated generative method for horizontally partitioned tabular data synthesis in multiple parties. In HT-Fed-GAN, the multimodal distribution in the decentralized tables extracted by the proposed Fed-VB-GMM can effectively eliminate mode collapse. To prevent private leakage, HT-Fed-GAN employs differential privacy during federated training rounds. To the best of our knowledge, this is the first work for privacy-preserving data synthesis on horizontally partitioned decentralized tabular datasets. We experimentally evaluated our HT-Fed-GAN on five real-world datasets from five different domains and showed were most statistically similar to the original tables without revealing private information. In terms of the performance of the machine learning tasks, HT-Fed-GAN outperformed the state-of-the-art model.

In the future, we plan to extend our method to vertically partitioned decentralized tables and further improve the data quality of the federated generative model.

## Figures and Tables

**Table 1:** Summary of notations.

Notation	Description
*x*	A variable of data.
$\pi_{k}$	The mixture weight of the *k*-th Gaussian distribution.
$N(x|\mu_{k},\Sigma_{k})$	The *k*-th Gaussian distribution with mean $\mu_{k}$ and variance $\Sigma_{k}$.
p(x|π,μ,Σ)	A Gaussian mixture model.
Dir(α)	A Dirichlet distribution with parameter α.
Λ	The inverse matrix of Σ.
W(W,ν)	A Wishart distribution with parameters *W* and ν.
$D^{{}^{\prime }}$	The neighboring dataset of *D*.
ϵ,δ	Parameters of a differential privacy algorithm.
*A*	A differential privacy algorithm.
ψ(.)	A digamma function.
Γ(.)	A gamma function.
$r_{j}$	The representation of the *j*-th record after normalization.
⊕	The concatenate operation.

**Table 2:** Statistics of Datasets.

Dataset	Number of Records	Number of Attributes	Classification Type	Attribute for Classification	Attribute for Regression	Split Attribute	Client	Number of Training Records	Values of Split Attributes	Number of Testing Records
Adult	32,561	15	binary classification	income	hours_per_week	education	Client 1	12,053	[‘Doctorate’, ‘Masters’, ‘Bachelors’, ‘Some-college’, ‘Assoc-acdm’, ‘Assoc-voc’]	9768
							Client 2	9551	[‘Prof-school’, ‘HS-grad’, ‘12th’, ‘11th’, ‘10th’]	
							Client 3	1189	[‘Preschool’, ‘1st–4th’, ‘5th–6th’, ‘7th–8th’, ‘9th’]	
Intrusion	494,021	41	multi-class classification	label	count	flag	Client 1	264,731	[’SF’]	148,206
							Client 2	61,235	[’S0’, ’SH’, ’S1’, ’S2’, ’S3’]	
							Client 3	19,849	[’REJ’,’RSTR’, ’RSTO’, ’RSTOS0’, ’OTH’]	
Credit	284,807	30	binary classification	label	Amount	Amount	Client-1	159,207	0∼100	85,442
							Client 2	38,001	100∼1000	
							Client 3	2157	1000∼20,000	
Cover-Type	581,012	55	multi-class classification	Cover_Type	Elevation	Elevation	Client-1	919	0∼2000	174,303
							Client 2	204,871	2000∼3000	
							Client 3	200,919	3000∼4000	
Health	299	13	binary classification	DEATH_EVENT	age	age	Client-1	31	0∼50	89
							Client 2	126	50∼70	
							Client 3	53	70∼100	

**Table 3:** Average F1 scores of binary classification tests on three datasets, where “-” represents mode collapse. The low-privacy setting indicates that differential privacy was not used in HT-Fed-GAN, whereas the high-privacy setting denotes that differential privacy was used in HT-Fed-GAN and the privacy budgets were ϵ = 0.5 and δ = 9.8.

Dataset	Origin Table	HT-Fed-GAN		DP-FedAvg-GAN	
		Low-Privacy	High-Privacy	Low-Privacy	High-Privacy
Adult	0.6711	**0.6067**	0.5886	0.3535	0.3595
Credit	0.5328	0.6860	**0.7179**	-	-
Health	0.6359	**0.6603**	0.5734	0.5723	0.4494

**Table 4:** Average macro- and micro-F1 scores of multi-classification tests. The low-privacy setting indicates that differential privacy was not used in HT-Fed-GAN, whereas the high-privacy setting denotes that differential privacy was used in HT-Fed-GAN and the privacy budgets were ϵ = 0.5 and δ = 9.8.

Dataset	Origin Table		HT-Fed-GAN (Low-Privacy)		HT-Fed-GAN (High-Privacy)		Dp-FedAvg-GAN (Low-Privacy)		Dp-FedAvg-GAN (High-Privacy)	
	Macro F1	Micro F1	Macro F1	Micro F1	Macro F1	Micro F1	Macro F1	Micro F1	Macro F1	Micro F1
Intrusion	0.8369	0.9990	**0.6151**	**0.9858**	0.5705	0.9765	0.3859	0.8559	0.1906	0.7928
Covetype	0.6158	0.7419	**0.4291**	**0.6513**	0.2826	0.5445	0.1444	0.4492	0.1100	0.4910

**Table 5:** Average MAE scores of regression tests, where “-” represents mode collapse. The low-privacy setting indicates that differential privacy was not used in HT-Fed-GAN, whereas the high-privacy setting denotes that differential privacy was used in HT-Fed-GAN and the privacy budgets were ϵ = 0.5 and δ = 9.8.

Dataset	Origin Table	HT-Fed-GAN		DP-FedAvg-GAN	
		Low-Privacy	High-Privacy	Low-Privacy	High-Privacy
Adult	0.2292	**0.2411**	0.2491	0.3931	0.3422
Credit	1.2383	**1.4844**	1.4852	-	-
Health	0.1869	0.3136	0.3400	**0.1990**	0.2467
Intrusion	0.2793	0.7522	**0.3946**	0.7697	1.1211
Cover-Type	0.0441	**0.0659**	0.0663	0.0754	0.1094

**Table 6:** F1 scores of membership inference attack, where "-" represents mode collapse. The low-privacy setting indicates that differential privacy was not used in HT-Fed-GAN, whereas the high-privacy setting denotes that differential privacy was used in HT-Fed-GAN and the privacy budgets were ϵ = 0.5 and δ = 9.8.

Dataset	HT-Fed-GAN		DP-FedAvg-GAN	
	Low-Privacy	High-Privacy	Low-Privacy	High-Privacy
Adult	0.511	0.4664	0.5615	0.5409
Credit	0.5666	0.4652	-	-
Health	0.5665	0.4832	0.578	0.5128
Intrusion	0.5328	0.4308	0.5645	0.456
Covertype	0.5667	0.4652	0.5625	0.6256

**Table 7:** Sample records in the synthesized table by HT-Fed-GAN with the low-privacy configuration.

	Age	fnlwgt	Eduction	Occupation	Hours_per_week	Income
1	33	173,520	7th–8th	Other-service	39	<=50 K
2	51	196,863	Doctorate	Prof-specialty	59	>50 K
3	47	254,381	HS-grad	Craft-repair	39	<=50 K
4	49	267,701	11th	Craft-repair	49	>50 K
5	28	198,232	HS-grad	Handlers-cleaners	39	<=50 K
6	29	164,833	HS-grad	Sales	43	>50 K

**Table 8:** Sample records in the original Adult table of each client.

	Age	fnlwgt	Eduction	Occupation	Hours_per_week	Income
1	22	48,988	Some-college	Transport-moving	40	<=50 K
2	55	296,085	Assoc-acdm	Prof-specialty	40	>50 K
3	23	170,070	12th	Other-service	38	<=50 K
4	40	178,983	HS-grad	Adm-clerical	30	>50 K
5	52	370,552	Preschool	Machine-op-inspect	40	<=50 K
6	36	328,466	5th–6th	Transport-moving	50	>50 K

## Abbreviations

The following abbreviations are used in this manuscript:

GAN	Generative Adversarial Network
GMM	Gaussian Mixture Model
VB-GMM	Variational Bayesian Gaussian Mixture Model
MAE	Mean Absolute Error
DP	Differential Privacy
